# Homelessness at discharge and its impact on psychiatric readmission and physician follow-up: a population-based cohort study

**DOI:** 10.1017/S2045796019000052

**Published:** 2019-03-07

**Authors:** V. Laliberté, V. Stergiopoulos, B. Jacob, P. Kurdyak

**Affiliations:** Centre for Addiction and Mental Health, University of Toronto, Toronto, ON, Canada

**Keywords:** Homeless persons, patient discharge, patient readmission, psychiatric hospitals

## Abstract

**Aims:**

A significant proportion of adults who are admitted to psychiatric hospitals are homeless, yet little is known about their outcomes after a psychiatric hospitalisation discharge. The aim of this study was to assess the impact of being homeless at the time of psychiatric hospitalisation discharge on psychiatric hospital readmission, mental health-related emergency department (ED) visits and physician-based outpatient care.

**Methods:**

This was a population-based cohort study using health administrative databases. All patients discharged from a psychiatric hospitalisation in Ontario, Canada, between 1 April 2011 and 31 March 2014 (*N* = 91 028) were included and categorised as homeless or non-homeless at the time of discharge. Psychiatric hospitalisation readmission rates, mental health-related ED visits and physician-based outpatient care were measured within 30 days following hospital discharge.

**Results:**

There were 2052 (2.3%) adults identified as homeless at discharge. Homeless individuals at discharge were significantly more likely to have a readmission within 30 days following discharge (17.1 *v.* 9.8%; aHR = 1.43 (95% CI 1.26–1.63)) and to have an ED visit (27.2 *v.* 11.6%; aHR = 1.87 (95% CI 1.68–2.0)). Homeless individuals were also over 50% less likely to have a psychiatrist visit (aHR = 0.46 (95% CI 0.40–0.53)).

**Conclusion:**

Homeless adults are at higher risk of readmission and ED visits following discharge. They are also much less likely to receive post-discharge physician care. Efforts to improve access to services for this vulnerable population are required to reduce acute care service use and improve care continuity.

## Introduction

Homeless people present with high rates of mental and physical illnesses (Hwang, 2001; Fazel *et al*., 2008, 2014). Rates of alcohol and drug dependence, as well as those of psychotic disorders and personality disorders, are especially high in homeless populations compared with the general population (Fazel *et al*., 2008). Homeless people are also afflicted by a wide number of physical illnesses, notably infectious diseases such as tuberculosis, hepatitis, HIV and sexually transmitted diseases (Hwang, 2001; Fazel *et al*., 2014). Chronic diseases, such as cardiovascular and metabolic diseases, are also common. The very high rate of tobacco use leads to frequent smoke-related diseases, such as chronic obstructive pulmonary disease, and age-related conditions, such as cognitive impairment, are increasing (Fazel *et al*., 2014). Rates of mortality are also much higher, which could be the consequence of the higher occurrence of unintentional injuries, of being victims of violence and of suicide (Hwang, 2001; Fazel *et al*., 2014). Moreover, people with mental illness in general present with a higher rates of numerous health problems compared with the general population, as a result of lifestyle and failure to receive adequate care when they are ill (De Hert *et al*., 2011). The lifespan of people with severe mental illness is reduced, and mortality rates of people with schizophrenia are threefold higher compared with those without schizophrenia (Gatov *et al*., 2017).

When homelessness and mental illness are combined, the burden of health problems is additive, with the consequence of higher health service utilisation. Homeless individuals with mental health problems consume mental health services at a rate that is nearly fourfold higher than the housed general population (Folsom *et al*., 2005). Despite these high needs, access to ambulatory health services is known to be low in this population. Homeless individuals with mental health problems are more likely to use acute care settings (hospitalisations (Chambers *et al*., 2013; Saab *et al*., 2016) and emergency departments (ED) (Kushel *et al*., 2002; Arfken *et al*., 2004)) and less likely to receive general primary care (Khandor *et al*., 2011) and outpatient mental health services (Folsom *et al*., 2005). However, little is known about the quality of care for homeless individuals discharged following a psychiatric hospitalisation to the street or to shelters (Burra *et al*., 2012), despite the fact that the month following discharge from a psychiatric ward is a period of high risk and high need (Dixon *et al*., 2009).

The Canadian definition of homelessness is ‘the situation of an individual, family or community without stable, permanent, appropriate housing, or the immediate prospect, means and ability of acquiring it’ (Gaetz *et al*., 2012). There are four types of homelessness: (1) unsheltered, (2) emergency sheltered, (3) provisionally accommodated and (4) at risk of homelessness (Gaetz *et al*., 2012). ‘Unsheltered’ describes individuals who are ‘absolutely homeless and living on the streets or in places not intended for human habitation’. This includes individuals who are living outside, for example, under bridges or in forests, and not only on the street. Emergency sheltered includes ‘those staying in overnight shelters for people who are homeless, as well as shelters for those impacted by family violence’ (Gaetz *et al*., 2012). Our definition includes the first two types of homelessness, which correspond to the most severe forms.

We used population-based health-administrative datasets in Ontario to compare homeless *v*. non-homeless individuals at psychiatric discharge, and to measure access psychiatric readmission following discharge as well as mental health-related ED visits. We also assessed visits to a psychiatrist or a family doctor following psychiatric discharge. We hypothesised that homeless adults at discharge would have greater illness severity and less access to follow-up care compared with non-homeless individuals, and that they therefore would have higher likelihood of psychiatric readmission to the hospital and of ED visits for mental health reasons. We also hypothesised that individuals discharged as homeless would have reduced likelihood of physician visits.

## Methods

### Study design and setting

This is a population-based cohort study using sociodemographic and health administrative data to measure the outcomes of homeless adults following discharge from a psychiatric hospitalisation in Ontario, Canada, between 2011 and 2014. All Ontario residents are covered by the Ontario Health Insurance Plan (OHIP), a universal, government-funded health insurance, which includes physician visits, psychiatric hospitalisations and ED visits.

In addition, there are also Ontario Ministry of Health and Long-Term Care-funded community-based services responsible for Active Community Treatment teams across the province. Since our study included individuals who have been discharged after a psychiatric hospitalisation, physicians are a necessary part of planned follow-up to ensure continuity of medication initiated or modified during the psychiatric hospitalisation and to provide medical assessment of stability and response to treatment. Indeed, receipt of a physician visit within 7 days of a hospitalisation discharge is a standard mental health system performance indicator routinely reported by Health Quality Ontario (Health Quality Ontario, 2018) as a measure of access to critical services at a time of high-risk transition from hospital to community settings.

### Data sources

Health administrative databases representing the population of Ontario were accessed at the Institute for Clinical Evaluative Sciences (ICES), an independent, non-profit research organisation that holds population-level data, including administrative data. A unique, encrypted identifier (ICES key number; IKN) is used to anonymously link the databases described below for each individual in the cohort. The databases used in this research can all be accessed through ICES. The Registered Persons Database (RPDB) contains information on age, gender and postal code (region of residence). Implemented in 2005, the Ontario Mental Health Reporting System (OMHRS) includes information on all admissions that occur in psychiatric inpatient beds for adults aged 18 and older in Ontario, which includes approximately 5000 psychiatric inpatient beds.

The data in the OMHRS are gathered using the Resident Assessment Instrument – Mental Health (RAI-MH) (Hirdes *et al*., 2000), a comprehensive clinical assessment tool first completed within 3 days of admission, capturing information such as the place of residence prior to admission, measures of psychiatric symptoms, the legal status of the admission, as well as aggressive behaviour. The RAI-MH is subsequently completed at 90-day intervals during the admission (where applicable) and at discharge, during which the place of residence is obtained. The Ontario Health Insurance Program (OHIP) database gathers data on all physicians and includes information on physician billings, including patient visits and diagnostic codes. The Canadian Institutes of Health Information – Discharge Abstract Database (CIHI-DAD) includes information obtained from non-psychiatric hospitalisations. The National Ambulatory Care Reporting System (NACRS) contains information on all ED visits. The Client Agency Program Enrolment (CAPE) database provides information on the type of primary care enrolment. Neighbourhood income quintile was based on the 2006 census data applied to 2011 census regions. The use of data in this project was authorised under section 45 of Ontario's Personal Health Information Protection Act, which does not require review by a Research Ethics Board.

### Cohort

This study included every patient discharged from a psychiatric hospitalisation in Ontario during a 3-year period: between 1 April 2011 and 31 March 2014. The patient's first discharge during that time period determined the index admission. Hospitalisations with a length of stay <72 h were excluded because they are missing key sources of information, including diagnosis (Urbanoski *et al*., 2012). Patients were excluded from the study if they had an invalid IKN, invalid or missing age or sex data, were younger than 16 or older than 105, died during the psychiatric hospitalisation, admitted for more than 365 days (our look back window would be exceeded), or transferred to another psychiatric hospital at the time of discharge. Homelessness was measured at discharge as part of the routine discharge assessment and was defined as individuals who live in shelters or on the streets.

### Outcomes

The primary outcomes were psychiatric readmission and mental health-related ED visits within 30 days following discharge. The secondary outcome of this study corresponds to any outpatient visits to a family physician, a psychiatrist or both, within the same time frame. The ‘early readmission’ outcome is recognised worldwide to indicate how patient needs are met in terms of coordination and continuity of services (Vigod *et al*., 2015). Moreover, timely follow-up care after hospitalisation is considered an important measure of the quality of mental health services (Stein *et al*., 2007). In Ontario, family doctors are much more numerous than psychiatrists and, therefore, are more likely to provide post-discharge follow-up care, particularly in rural areas (Chiu *et al*., 2018).

### Covariates

The sociodemographic variables included age, sex and rurality (community that has fewer than 10 000 residents), living situation at admission, as well as income quintile. Clinical variables included the Depression Rating Scale (DRS), Positive Symptom Scale (PSS) and Mania Symptom Scale (MSS), all captured in the RAI (Hirdes *et al*., 2002). We also measured the presence of involuntary status and used the Aggressive Behavior Scale as proxy for illness severity (Martin and Hirdes, 2009). We estimated the overall medical comorbidity using the Johns Hopkins Aggregated Diagnosis Groups covering a 2-year period (ADGs). The Johns Hopkins ADGs are a method of ascertaining medical comorbidities and have been validated as predictors of mortality in the general population and for individuals with schizophrenia (Starfield *et al*., 1991; University John Hopkins). The prior health service utilisation category included both mental health (primary care visits for mental health conditions, outpatient psychiatrist visits, psychiatric hospitalisations and ED) and non-mental health variables (primary care visits and ED) (Steele *et al*., 2004). Finally, we measured whether patients were rostered to a primary care physician prior to the index admission.

### Statistical tests

First, bivariate association between potential sociodemographic, clinical and prior service utilisation predictors, and the main exposure (homeless at discharge) were assessed using *t*-tests for continuous variables and *χ*^2^ for dichotomous or categorical variables (see Table 1). Second, we assessed the associations between our main exposure and our primary and secondary outcomes, also using *χ*^2^ tests (Table 2). Finally, we measured the association between our sociodemographic and clinical predictors and our main outcomes: readmission and ED visits at 30 days (Table 2), using Cox Proportional Hazard survival time to event analysis (for full Cox Proportional Hazard models, see online Supplementary Tables 1a–c). We also used Cox Proportional Hazard survival analysis for outpatient care at 30 days (a secondary outcome). There were no violations of the assumption of proportionality for all Cox Proportional Hazard models reported in this study. Analyses were conducted using SAS version 9.4.

**Table 1.** Baseline characteristics of patients at admission who were homeless at discharge

Variable	Homeless at discharge (*n* = 2052)	Not-homeless at discharge (*n* = 88 976)	Statistics (standardised difference)
Demographics			
Age at index date, median (IQR)	37.69	41.94	0.27
Female gender, *n* (%)	726 (35.4)	44 237 (49.7)	0.29
Income quintile, *n* (%)			
1	710 (34.6)	25 176 (28.3)	0.14
2	417 (20.3)	18 773 (21.1)	0.02
3	333 (16.2)	15 790 (17.7)	0.04
4	316 (15.4)	15 432 (17.3)	0.05
5	227 (11.1)	13 178 (14.8)	0.11
Missing	49 (2.4)	667 (0.7)	0.13
Rural residence, *n* (%)	100 (4.9)	10 053 (11.3)	0.24
Homeless at admission	1247 (60.8)	1823 (2)	1.63
Clinical characteristics			
Involuntary status, *n* (%)	1550 (75.5)	58 846 (66.1)	0.21
Aggressive Behavior Scale (range 0–12) mean, std	1.1 (2.1)	0.46 (1.4)	0.36
Depression Rating Scale (range 0–15) mean, std	1.8 (2.1)	1.63 (2.1)	0.08
Mania scale (0–18) mean, std	2.32 (3.3)	1.35 (2.6)	0.33
Positive Symptoms Scale (long) mean, std	2.18 (3.4)	1.34 (2.7)	0.28
Comorbidities (ADGs) mean, std	5.4 (3.8)	5.67 (3.6)	0.07
Prior service utilisation (1-year prior to index admission)			
Rostered to a physician, *n* (%)	1802 (87.8)	84 041 (94.5)	0.24
Family physician visits, mean, std	7.24 (13)	7.37 (10.6)	0.01
0 *n*(%)	496 (24.2)	12 681 (14.3)	0.25
1	220 (10.7)	8865 (10)	0.02
2	189 (9.2)	8233 (9.3)	0
3 +	1147 (55.9)	59 197 (66.5)	0.22
Family physician visits (for non-mental health conditions)	5.59 (13.5)	5.71 (11.2)	0.01
0	669 (32.6)	19 556 (22.0)	0.24
1	296 (14.4)	12 390 (13.9)	0.01
2	212 (10.3)	10 290 (11.6)	0.04
3 +	875 (42.6)	46 740 (52.5)	0.2
Family physician visits (for mental health conditions)	2.56 (5.5)	2.43 (4.9)	0.02
0	940 (45.8)	37 399 (42)	0.08
1	344 (16.8)	16 345 (18.4)	0.04
2	205 (10.0)	9914 (11.1)	0.04
3 +	563 (27.4)	25 318 (28.5)	0.02
Outpatient psychiatrists visits	2.55 (10.5)	2.83 (6.9)	0.03
0	1170 (57)	50 923 (57.2)	0
1	248 (12.1)	8811 (9.9)	0.07
2	133 (6.5)	5005 (5.6)	0.04
3 +	501 (24.4)	24 237 (27.2)	0.06
Number of previous psychiatric hospitalisations	0.36 (1.2)	0.22 (0.8)	0.14
0	1700 (82.8)	76 182 (85.6)	0.08
1	210 (10.2)	9373 (10.5)	0.01
2	64 (3.1)	1991 (2.2)	0.05
3 +	78 (3.8)	1430 (1.6)	0.14
Number of ED visits	4.43 (7.9)	2.38 (4.2)	0.32
0	383 (18.7)	24 231 (27.2)	0.2
1	458 (22.3)	24 268 (27.3)	0.11
2	305 (14.9)	14 501 (16.3)	0.04
3 +	906 (44.2)	25 976 (29.2)	0.31
Number of ED visits for psychiatric conditions	1.93 (4.6)	0.83 (2)	0.31
0	837 (40.8)	49 186 (55.3)	0.29
1	594 (28.9)	25 610 (28.8)	0
2	246 (12.0)	7810 (8.8)	0.11
3 +	375 (18.3)	6370 (7.2)	0.34

IQR, interquartile range; STD, standard deviation; ADGs, Aggregated Diagnosis Groups; ED, emergency department.

**Table 2.** Outcomes after discharge according to the living situation

	Homeless at discharge *n* = 2052	Not-homeless at discharge *n* = 88 976	Statistics (*p* value)	Unadjusted HR (95% hazard ratio confidence limits)	Adjusted HR (95% hazard ratio confidence limits)
Primary outcomes					
Readmission within 30 days of discharge					
Psychiatric readmission	350 (17.1)	8685 (9.8)	<0.001	1.84 (1.65–2.05)	1.43 (1.26–1.63)
Mental health-related ED visits	559 (27.2)	10 313 (11.6)	<0.001	2.64 (2.43–2.88)	1.87 (1.68–2.08)
Secondary outcomes					
Outpatient care within 30 days of discharge (any reasons)					
No family physician or psychiatrist visit	950 (46.3)	28 394 (31.9)	<0.001	–	–
Family physician visit only	549 (26.8)	28 124 (31.6)	<0.001	0.67 (0.62–0.73)	0.88 (0.8–0.97)
Psychiatrist visit only	329 (16)	17 151 (19.3)	<0.001	0.64 (0.57–0.71)	0.46 (0.4–0.53)
Family physician and psychiatrist visit	224 (10.9)	15 307 (17.2)	<0.001	0.49 (0.43–0.56)	0.47 (0.40–0.56)
Outpatient care within 30 days of discharge (for mental health reasons)					
No family physician or psychiatrist visit	1160 (56.5)	38 433 (43.2)	<0.001	–	–
Family physician visit only	339 (16.5)	18 085 (20.3)	<0.001	–	–
Psychiatrist visit only	402 (19.6)	22 628 (25.4)	<0.001	–	–
Family physician and psychiatrist visit	151 (7.4)	9830 (11.0)	<0.001	–	–

ED, emergency department.

## Results

There were 95 230 patients discharged from a psychiatric inpatient unit during the study period. After applying our exclusion criteria, 91 028 patients remained in the study cohort. Of those, 2052 patients (2.3%) were identified as homeless at discharge. Baseline characteristics by homeless status at discharge are found in Table 1. Homeless adults at discharge were more likely to be male, younger, to reside in lower-income neighbourhoods and in urban settings (see Table 1). Patients who were homeless at discharge had higher rates of involuntary hospitalisation and higher aggressive behaviour, depression, mania and positive psychotic symptoms scale scores. However, they had less documented medical comorbidity. In the year preceding their admission, they were less likely to be rostered to a physician and to have had primary care visits, mental health-related or not, or psychiatry visits. During that same year, they were much more likely to have had psychiatric hospitalisations and ED visits for both mental health and non-mental health reasons.

**Fig. 1.**
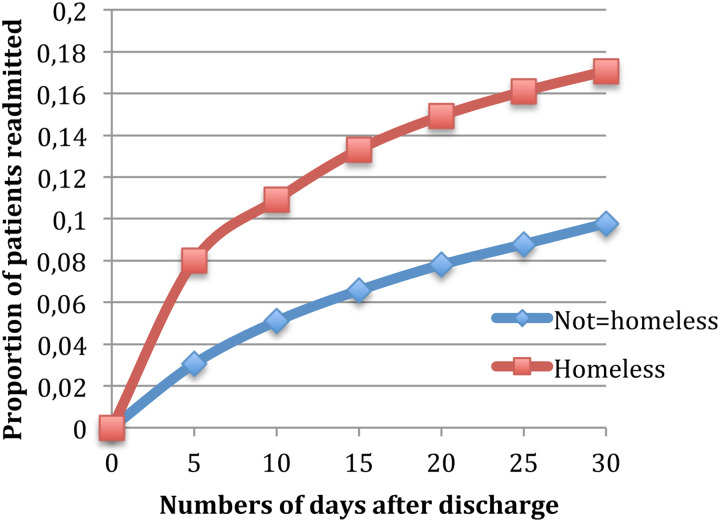
Readmission rates within 30 days of discharge.

The risk of a psychiatric readmission at 30 days was 17.1% for homeless patients, in comparison with 9.8% for non-homeless patients (aHR  = 1.43 (95% CI 1.26–1.63)) (see Fig. 1). Finally, being homeless at discharge increased the risk of having a mental health-related ED visits at 30 days nearly twofold (27.2 *v*. 11.6%, 95% CI; aHR  = 1.87 (95% CI 1.68–2.08)).

In terms of follow-up care after discharge for any reasons (mental health-related or others), 46.3% of the homeless population had no outpatient care in the 30 days that followed discharge (46.3 *v*. 28.4%, *p* < 0.0001) (see Table 2). Homeless individuals were less likely to see a family physician (26.8 *v.* 31.6%, aHR  = 0.88 (95% CI 0.80–0.97)), a psychiatrist (16.0 *v.* 19.3%, aHR  = 0.463 (95% CI 0.40–0.53)) or both a family physician and a psychiatrist (10.9 *v*. 17.2%, aHR  =  0.471 (95% CI 0.40–0.56)). When only mental health-related visits post-discharge were considered, 56.5% of homeless patients had no outpatient care in the same period (56.5 *v*. 43.2%, *p* < 0.0001). Homeless adults were also less likely to have had a mental health-related family physician visit (16.5 *v*. 20.3%, *p* < 0.001), an outpatient psychiatry visit (19.6 *v*. 25.4%, *p* < 0.001) or both (7.4 *v*. 11.0%, *p* < 0.001).

## Discussion

To our knowledge, this is the first study to evaluate outcomes following discharge in a large comprehensive population dataset of homeless adults with mental illness. This is the first study to date to examine the rate of readmission within specific periods of time following discharge from a psychiatric hospital, and by far the largest study of outpatient visits after psychiatric discharge. At the time of discharge from a psychiatric hospitalisation, more than one out of every 50 adult patients was identified as homeless. The reality of psychiatric discharge to the street or to shelter has been rarely explored (Forchuk *et al*., 2006). Patients with mental illness often feel that their housing needs are not taken into account sufficiently at discharge from a psychiatric hospital (Drury, 2008), despite the evidence that proper discharge planning is critical to avoid homelessness (Backer *et al*., 2007). Shelters are not appropriate places to recover from an episode of mental illness requiring hospitalisation (Forchuk *et al*., 2006).

Compared with their housed counterparts, homeless people at discharge tended to be men, younger and to have higher illness severity. In the year prior to their psychiatric admission, individuals discharged as homeless were less likely to have had family physician visits or outpatient psychiatry visits, while at the same time more likely to have used acute care services: psychiatric hospitalisation and ED visit. They were also less likely to have physician visits either from a primary care physician or from a psychiatrist within 30 days following discharge, suggesting that homelessness is associated with paradoxically poor access to care following discharge despite higher need for care continuity based on illness severity. During that same period of time, homeless adults were also 43% more likely to be readmitted to a psychiatric unit. These findings point to the importance of optimizing the transition to outpatient care following discharge from hospital. For example, the Critical Time Intervention model has been shown to decrease homelessness (Herman *et al*., 2011) and psychiatric readmission after hospital discharge in New York City (Tomita and Herman, 2012), as well as in Europe (de Vet *et al*., 2017). In this model, a Critical Time Intervention worker provides a time-limited, strength-based intervention that aims to bridge the gap between services during period of transition by providing practical and emotional support and by connecting the person to community resources (de Vet *et al*., 2017). Findings also highlight the need for programmes that directly address housing issues, such as the At Home/Chez Soi project in Canada, which used the ‘Housing First’ approach with mental health support services (Goering *et al*., 2014; Stergiopoulos *et al*., 2015), and was showed to increase housing stability over 24 months (Stergiopoulos *et al*., 2015). The Housing First approach originated in the USA and is now present in many European countries, notably in Finland where it had a positive impact.

Previous studies have reported high readmission rates for homeless adults after a hospital discharge (Appleby and Desai, 1987; Lay *et al*., 2006; Irmiter *et al*., 2007; Schmutte *et al*., 2010; Hamilton *et al*., 2015; Lorine *et al*., 2015), especially within 30 days. For example, the nine homeless individuals included in the seminal study done by Appleby and Desai (1987) all tended to be ‘chronic recidivists’. In terms of psychiatric readmission, prior living on the street or in a homeless shelter was shown to predict readmission at 30 and 90 days in a study that included 2443 adults consecutively admitted in a public psychiatric hospital for bipolar affective disorder (Hamilton *et al*., 2015). In another study that included 424 first admitted psychotic patients, homelessness was found to be an important predictor of being a ‘heavy’ or ‘frequent’ user (at least 300 days in the hospital or more than three psychiatric admissions) (Lay *et al*., 2006). Homelessness was also the strongest predictor of ‘time to re-hospitalisation’ in a large study of 35 527 patients hospitalised in psychiatry in the Department of Veterans Affairs Health system (Irmiter *et al*., 2007). Nevertheless, homelessness has not been consistently associated with high readmission rates following psychiatric hospitalisation discharge in two studies that contained a limited number of homeless people, which may explain the inconsistent findings (Casper, 1995; Schmutte *et al*., 2009). In terms of acute care services use, we also found that homelessness at discharge predicts an 83% higher risk of mental health-related ED visit in the 30 days following discharge. This is in agreement with previous studies (Arfken *et al*., 2004; Pasic *et al*., 2005). Homeless people are known to be high users of the ED, and these visits are often the consequence of a psychiatric condition, notably substance abuse (Capp *et al*., 2013; Tsai and Rosenheck, 2013). These findings point to the importance of optimizing the transition to outpatient care following discharge from hospital, through programmes such as the Critical Time Intervention (Herman *et al*., 2011; Tomita and Herman, 2012) or At Home/Chez Soi in Canada, which used the ‘Housing First’ approach with mental health support services (Stergiopoulos *et al*., 2015).

Continuity of care following a psychiatric discharge is known to be low (Boyer, 1997). A study conducted in the USA, for example, showed that only 49% of patients received follow-up care in the 30 days after a psychiatric discharge (Stein *et al*., 2007). Only one study in Canada examined continuity of care in the homeless population following a psychiatric discharge, in a cohort of 30 homeless patients with schizophrenia and schizo-affective disorder (Burra *et al*., 2012). These participants were less likely than the 21 housed controls to have follow-up appointments with a family physician, or to access intensive case management or assertive community treatment. The fact that homeless individuals were less likely to be rostered to a family physician and less regularly followed by primary care physician or a psychiatrist prior to the index admission (see Table 1) is also congruent with prior research: for example, Khandor *et al*. (2011) showed that less than half of homeless people in Ontario's largest urban centre reported having a family doctor, despite a system of universal health insurance. Organisational barriers to accessing care in the homeless population, such as lack of identifying documents and cost of medication, attitudinal barriers such as stigma and discrimination, in addition to competing priorities and the chaotic lifestyles secondary to mental illness and substance misuse, may lead this population to experience health services as fragmented and interrupted (Canavan *et al*., 2012; Skosireva *et al*., 2014; Campbell *et al*., 2015; Bradley, 2018). Lower continuity of care may also be attributed to high rates of missed appointments, or ‘no-shows’. Individuals with severe mental illnesses are known to be more difficult to engage and to have high dropout rates (Kreyenbuhl *et al*., 2009; Dixon *et al*., 2016), and disengagement rates would even be higher among mentally ill individuals with ‘low social functioning’ (Kreyenbuhl *et al*., 2009). Substance use, homelessness and unemployment were also associated with ‘no-shows’ in a gastroenterology clinic (Chang *et al*., 2015). The establishment of resources (e.g. brief case management following discharge) to ensure homeless individuals have a stable transition following discharge may be necessary to reduced readmission rates.

### Limitations

One limitation of this research is that the indicator of homelessness at discharge found in the OMHRS dataset has not been validated, hence some participants might have been assigned the wrong housing status (Tsai *et al*., 2005). Homelessness is likely under-reported which would result in an underestimate of the prevalence of homelessness in our study population (Susser *et al*., 1997; Tsai *et al*., 2005). However, our indicator of being homeless is distinct and potentially more precise than other studies because it measures the housing status directly at discharge. In contrast, most of the studies considering psychiatric readmissions of homeless people that we reviewed did their analysis considering homelessness at admission (Lay *et al*., 2006; Hamilton *et al*., 2015; Lorine *et al*., 2015), which is problematic because an admission is known to be a period of high residential mobility, especially in the homeless population (Tsai *et al*., 2005; Tulloch *et al*., 2011). Another study based the homeless status on a Fiscal Year report (Irmiter *et al*., 2007), or on chart review without further specifications (Schmutte *et al*., 2010). Hence even though it is likely that there were some false negatives among our non-homeless sample, it appears very unlikely that we have false-positive homeless cases given the low likelihood of someone discharged to housing being classified as homeless. Considering the Canadian definition of homelessness reported earlier, our data do not capture individuals who are provisionally accommodated and those who are at risk of homelessness. These groups often referred to as ‘hidden homelessness’, may be the largest and often fall below the radar despite potentially having important mental health needs. Another limitation is that the OMHRS dataset did not allow us to separate individuals who are discharged to shelters from those discharged to the street, who might present different characteristics and outcomes. The fact that homelessness is defined differently across studies limits comparisons and attempts are being made to create common terminology (Tsai *et al*., 2005; Fazel *et al*., 2014). Another limitation of our study is that we were interested in physician visits after discharge and did not take into account other services that patients can receive, for example, from other professionals such as nurse, social workers and other mental health workers working in different settings including community organisations. However, physician visits remain a necessary, if not sufficient, marker of continuity of care for two reasons. First, patients discharged from a psychiatric hospital, whether homeless or not, are still relatively unstable and they require ongoing clinical monitoring by a physician about their treatment in order to evaluate and respond to clinical issues post-discharge. Second, patient will need a renewal of a medication prescription, and are hence expected to see a doctor as part of the discharge planning. Furthermore, although community services are important, it is not clear that they are preferentially available to homeless individuals, and if they are, they do not have an impact on the readmission rate and mental health-related emergency visits (our primary outcome) observed amongst the homeless population. Information about ‘no-shows’ would be useful in order to determine the importance of this factor to explain lower physician visits rates in the homeless at discharge population. Moreover, while we had access to a substantial amount of clinical information (e.g. numerous clinical rating scales), we did not have access to clinical information known to be both common among homeless patients and to likely influence outcomes such as substance use. We focused on homelessness as a key determinant to access to care in this research, even though there are a variety of barriers to mental health care as we mentioned in the discussion. Homelessness is indeed a major factor at the root of all those barriers. We also did not measure social supports (family supports, community and social agency supports) available to patients following discharge (Dyck *et al*., 2002). It would also be useful to assess the role of medication adherence in subsequent use of health services among people discharged as homeless. Future studies could further precise the relationship between having access to stable housing and adherence to a discharge planning that usually include follow-up visits. It would also be useful to obtain information about outcomes after discharge of patients who have a brief stay in the hospital (<72 h). In the future, studies could also attempt to characterise the specific mental health needs of individuals facing ‘hidden homelessness’.

Homeless has become a crisis worldwide and people facing it have significant unmet mental health needs (Gaetz *et al*., 2016; FEANTSA, 2017). Homelessness at discharge is an important predictor of recurrent use of acute mental health care services as well as discontinuity of care. Interventions that promote an efficient transition to outpatient care, or that address housing needs, may be necessary to reduce the rates of readmission after discharge.

## Supplementary material

For supplementary material accompanying this paper visit http://dx.doi.org/10.1017/S2045796019000052. click here to view supplementary material

## Availability of data and materials

All data supporting our study can be found in the ICES databases.
