# Supplementary material for: Homelessness at discharge and its impact on psychiatric readmission and physician follow-up: a population-based cohort study
Source: Epidemiol Psychiatr Sci. 2019 Mar 7;29:e21. doi: 10.1017/S2045796019000052 (PMC8061292; doi:10.1017/S2045796019000052)
Supplement: Supplementary file 1 [file S2045796019000052sup001.docx]

Supplementary Table 1a: Cox Proportional Hazard model of receiving family physician care only within 30-day of discharge.

| **Parameter** | **Hazard Ratio** | **95% Hazard Ratio Confidence** | |
| --- | --- | --- | --- |
|  |  | **Limits** | |
| Homeless at discharge | 0.880 | 0.800 | 0.968 |
| Age at index date | 1.007 | 1.006 | 1.007 |
| Male gender | 0.972 | 0.949 | 0.996 |
| Income quintile 1 | REF | - | - |
| Income quintile 2 | 1.101 | 1.065 | 1.139 |
| Income quintile 3 | 1.156 | 1.116 | 1.197 |
| Income quintile 4 | 1.194 | 1.153 | 1.237 |
| Income quintile 5 | 1.180 | 1.136 | 1.226 |
| Rural residence | 0.914 | 0.882 | 0.947 |
| Homeless at admission | 0.804 | 0.742 | 0.871 |
| Involuntary status | 0.912 | 0.889 | 0.934 |
| Aggressive behaviour scale | 0.984 | 0.975 | 0.994 |
| Depression rating scale | 1.015 | 1.009 | 1.021 |
| Mania scale | 1.007 | 1.000 | 1.014 |
| Positive symptoms scale (long) | 0.979 | 0.973 | 0.985 |
| Comorbidities (ADGs) | 1.052 | 1.048 | 1.056 |
| Rostered to a physician | 3.135 | 2.882 | 3.411 |
| Family phisician visits (for mental health conditions) | 1.032 | 1.03 | 1.033 |
| Outpatient psychiatrists visits | 0.983 | 0.980 | 0.987 |
| Psychiatric hospitalisations | 0.928 | 0.909 | 0.947 |
| ED visits (for mental health conditions) | 0.993 | 0.986 | 1.000 |

Abbreviations:

ADGs: Aggregated Diagnosis Groups

ED: emergency department

Supplementary Table 1b: Cox Proportional Hazard model of receiving psychiatric care only within 30-day of discharge.

| **Parameter** | **Hazard Ratio** | **95% Hazard Ratio Confidence** | |
| --- | --- | --- | --- |
|  |  | **Limits** | |
| Homeless at discharge | 0.463 | 0.401 | 0.534 |
| Age at index date | 0.998 | 0.997 | 0.999 |
| Male gender | 0.829 | 0.804 | 0.855 |
| Income quintile 1 | REF | - | - |
| Income quintile 2 | 1.106 | 1.059 | 1.155 |
| Income quintile 3 | 1.176 | 1.123 | 1.230 |
| Income quintile 4 | 1.233 | 1.178 | 1.290 |
| Income quintile 5 | 1.330 | 1.268 | 1.394 |
| Rural residence | 0.576 | 0.544 | 0.609 |
| Homeless at admission | 0.908 | 0.826 | 0.999 |
| Involuntary status | 0.921 | 0.892 | 0.952 |
| Aggressive behaviour scale | 0.949 | 0.937 | 0.962 |
| Depression rating scale | 0.993 | 0.985 | 1.002 |
| Mania scale | 0.976 | 0.967 | 0.984 |
| Positive symptoms scale (long) | 1.037 | 1.029 | 1,044 |
| Comorbidities (ADGs) | 0.994 | 0.989 | 0.999 |
| Rostered to a physician | 1.324 | 1.246 | 1.408 |
| Family phisician visits (for mental health conditions) | 1.010 | 1.006 | 1.013 |
| Outpatient psychiatrists visits | 1.016 | 1.016 | 1,017 |
| Psychiatric hospitalisations | 1.001 | 0.981 | 1.021 |
| ED visits (for mental health conditions) | 1.010 | 1.004 | 1.017 |

Abbreviations:

ADGs: Aggregated Diagnosis Groups

ED: emergency department

Supplementary Table 1c: Cox Proportional Hazard model of being seen by both a family physician and a psychiatrist within 30-day of discharge.

| **Parameter** | **Hazard Ratio** | **95% Hazard Ratio Confidence** | |
| --- | --- | --- | --- |
|  |  | **Limits** | |
| Homeless at discharge | 0.471 | 0.398 | 0.557 |
| Age at index date | 1.005 | 1.004 | 1.006 |
| Male gender | 0.841 | 0.813 | 0.869 |
| Income quintile 1 | REF | - | - |
| Income quintile 2 | 1.157 | 1.104 | 1.212 |
| Income quintile 3 | 1.201 | 1.144 | 1.261 |
| Income quintile 4 | 1.298 | 1.237 | 1.363 |
| Income qui | 1.405 | 1.337 | 1.476 |
| Rural residence | 0.51 | 0.478 | 0.545 |
| Homeless at admission | 0.736 | 0.653 | 0.83 |
| Involuntary status | 0.925 | 0.894 | 0.956 |
| Aggressive behaviour scale | 0.952 | 0.939 | 0.965 |
| Depression rating scale | 1.016 | 1.008 | 1.024 |
| Mania scale | 0.996 | 0.987 | 1.005 |
| Positive symptoms scale (long) | 1.01 | 1.002 | 1,019 |
| Comorbidities (ADGs) | 1.067 | 1.063 | 1.072 |
| Rostered to a physician | 3.718 | 3.269 | 4.227 |
| Family phisician visits (for mental health conditions) | 1.031 | 1.03 | 1.033 |
| Outpatient psychiatrists visits | 1.016 | 1.015 | 1,016 |
| Psychiatric hospitalisations | 0.929 | 0.907 | 0.952 |
| ED (mental health) visits | 1.006 | 0.999 | 1.012 |

Abbreviations:

ADGs: Aggregated Diagnosis Groups

ED: emergency department
